# Beyond hemoglobin: uncovering iron deficiency and iron deficiency anemia using serum ferritin concentration among pregnant women in eastern Ethiopia: a community-based study

**DOI:** 10.1186/s40795-022-00579-8

**Published:** 2022-08-17

**Authors:** Meseret Belete Fite, Demiraw Bikila, Wossene Habtu, Abera Kenay Tura, Tesfaye Assebe Yadeta, Lemessa Oljira, Kedir Teji Roba

**Affiliations:** 1 Department of Public Health, Institute of Health Sciences, Wollega University, Nekemte, Ethiopia; 2 Department of National Clinical Chemistry Reference Laboratory, Ethiopian Public Health Institute, Addis Ababa, Ethiopia; 3 School of Nursing and Midwifery, College of Health and Medical Sciences, Haramaya University, Harar, Ethiopia; 4 Department of Obstetrics and Gynaecology, University Medical Centre Groningen, University of Groningen, Groningen, the Netherlands; 5 School of Public Health, College of Health and Medical Sciences, Haramaya University, Harar, Ethiopia

**Keywords:** Serum ferritin, iron deficiency, iron deficiency anemia, Eastern Ethiopia

## Abstract

**Background:**

Although the high burden of anemia among pregnant women in low-resource settings like Ethiopia is well documented, evidence is scarce on the underlying causes using biochemical tests. Therefore, this study assessed the iron status and factors associated with iron deficiency (ID) using serum ferritin concentration among pregnant women in Haramaya district, eastern Ethiopia.

**Methods:**

A community-based cross-sectional study was conducted among randomly selected pregnant women in Haramaya district, eastern Ethiopia. The serum ferritin (SF) concentration was measured in the National Biochemical Laboratory of Ethiopia on a fully automated Cobas e411 (German, Japan Cobas 4000 analyzer series) immunoassay analyzer using the electro-chemiluminescence (ECL) method and standard procedures. A log-binomial regression analysis identified variables associated with iron deficiency, and defined as serum ferritin concentration < 15 μg/L (per the World Health Organization recommendation in developing countries). An adjusted risk ratio (aRR), and a 95% confidence interval (CI), were used to report associations. Finally, the *p*-value < 0.05 was the cut-off point for the significant association.

**Results:**

A total of 446 pregnant women with a mean age of 24.78 (+ 5.20) were included in the study. A total of 236 (52.91%; 95% CI: 48.16–57.63) had iron deficiency. The overall prevalence of anemia and iron deficiency anemia (IDA) was 45.96% (95% CI: 41.32–50.71) and 28.03% (95% CI: 21.27–32.44), respectively. The risk of iron deficiency was more likely among women with low dietary diversity (aRR = 1.36; 95% CI = 1.07–1.72) and those who skipped meals (aRR = 1.29; 95% CI = 1.05–1.57), but less among women who had antenatal care (aRR = 0.73 (95% CI = 0.61–0.88).

**Conclusion:**

More than half of the pregnant women in eastern Ethiopia had iron deficiency. Improving dietary diversity, meal frequency, and prenatal follow-up is essential to improve the high burden of ID and the adverse effect on pregnant women and the fetus. Moreover, a prospective study comparing maternal and perinatal outcomes among these spectra—iron depletion, ID, and IDA—is crucial for understanding their impact on maternal and perinatal mortality and morbidity.

## Introduction

The iron in the body is tightly regulated and dependent on nutritional demands and availability [1]. Because iron is crucial for physiological processes, including hemoglobin (Hb) synthesis, and cell growth and development [2], pregnant women have a greater demand for iron due to physiological increases in blood volume and needs [3]. Although the increased iron demand during pregnancy is believed to be met through cessation of menstrual losses, elevated intestinal absorption, and mobilization of maternal iron stores [4], a high proportion of pregnant women have low pre-pregnancy iron stores [5, 6], putting them at an increased risk of ID. As such, iron deficiency (ID) results from depletion of stored iron if not adequately replaced, resulting in iron deficiency anemia (IDA) [7]. According to the World Health Organization estimate (WHO) [8], serum ferritin levels less than 15 μg/L (throughout all trimesters of pregnancy) indicate ID [4]. IDA represents an array ranging from iron depletion without anemia (reduced iron stores with normal Hb concentration) to overt anemia, where the iron provision is inadequate to keep within normal Hb concentrations [9]. In addition, iron deficiency affects children’s psychological and physical development, weakens immunity, and increases pregnancy-related complications, prematurity and low birth weight [10–14].

WHO estimate, 30–40% of pregnant women are iron deficient, of which approximately half are anemic [15]. Furthermore, recent evidence suggested that ID among pregnant women was 20% in Australian [16], 20% in [17] in Malawi, 70% in China [18] and 76.7% Pakistan [19]. Using Hb levels for assessing iron deficiency among pregnant women at the population level, which is standard practice, may not reveal the problem since Hb can be low due to other causes. Additionally, the increase in plasma volume reduces Hb concentration, regardless of iron status [20, 21]. For these reasons, serum concentration of iron will better reveal the actual burden of iron depletion and related anemia. Because it is critical to determine body iron status in pregnancy, ferritin is the most commonly used index [15]. Serum ferritin (SF) is the most practical and sensitive screening test for iron accumulation during pregnancy and has been recommended as the screening test [22]. However, most existing studies, especially from low resource settings, do not use SF to estimate anemia; using other techniques without measuring SF [6], may not reveal the true clinical or public health burden [15]. In this study, we assessed maternal iron status using SF and examined the proportion of ID among pregnant women in Haramaya District, Eastern Ethiopia.

## Methods

### Study settings

The study was embedded into the Haramaya Health Demographic Surveillance and Health Research Centre (HDS-HRC), established in 2018. The HDS-HRC covers 12 rural kebeles (the lowest administrative unit in Ethiopia) out of 33 found in the district located approximately 500 KM from the capital city, Addis Ababa. Of 5252 pregnant women in the district during the study period, 2306 were followed by the HDS-HRC [23, 24]. This study was conducted from January 5 to February 12, 2021.

### Study design and population

A community-based cross-sectional study was conducted with all pregnant women in the district as the source population. In contrast, pregnant women who lived in randomly selected kebeles for at least 6 months during the study period were the study population. The sample size was determined using single and double population proportion formulas with their corresponding assumption, and the largest sample size was considered. As such, the sample was computed using the single population proportion formula with the following assumptions: 95% confidence interval, the prevalence of ID among pregnant women in Sidama Zone (33%) [25], 5% marginal error, and 10% non-response rate; the final computed sample size was 375. However, since this study was part of a larger longitudinal study (a prospective cohort study aimed to assess neonates’ birth weight and the association with maternal iron status), the same 475 pregnant women were included.

### Data collection

Data were collected through face-to-face interviews, anthropometric measurement, and serum ferritin analysis by trained research assistants. The questionnaire contained data on socio-economic, obstetric, maternal perception, food consumption, dietary diversity, knowledge, attitude, and practices of pregnant women. In addition, mid-upper arm circumference (MUAC) and maternal height measurements were taken. The questionnaire was initially prepared in English and translated to the local language (Afan Oromo) by individuals with good command of both languages. It was also pre-tested on 10% of the samples in Kersa District before actual implementation.

The formerly validated food frequency questionnaire (FFQ) containing 27 of the most common lists of food items consumed by the district community was used to assess the dietary diversity of the study participants [26–31]. The food items in the FFQ were grouped into ten food groups, including cereal, white roots and tubers, pulse and legumes, nuts and seeds, dark green leafy vegetables, other vitamin A-rich fruits and vegetables, meat, fish and poultry, dairy and dairy product, egg, other vegetables, and other fruits. The sum of each food group pregnant women consumed over 7 days was calculated to analyze the dietary diversity scores (DDS) [30]. Furthermore, the dietary diversity score was converted into tertiles, with the highest tertile labeled as a “high dietary diversity score” whereas both lower tertiles combined were defined as a “low dietary diversity score”. The food variety score (FVS) is the frequency of individual food items consumed during the reference period. Therefore, it was estimated by calculating each individual’s intake of the 27 food items over 7 days. A detailed description has been given elsewhere in the previous paper [32].

### Blood sample collection, serum extraction, and ferritin level determination

A 5 ml venous blood sample was aseptically drawn from the antecubital veins into plain test tubes without anticoagulants. The blood samples were centrifuged, followed by separation of serum, stored at − 80 °C, and later analyzed at the National Chemistry Laboratory in Ethiopian Public Health Institute (EPHI). We measured SF and serum high-sensitive C-reactive protein (hsCRP). SF was analyzed on a fully automated Cobas e411 (German, Japan Cobas 4000 analyzer series) immunoassay analyzer using the electro-chemiluminescence (ECL) method and commercial kits supplied by Roche Company, Germany, at National Clinical Chemistry Reference Laboratory, EPHI. At the same time, highly sensitive C-reactive protein (hsCRP) was analyzed by the Roche/Hitachi Cobas 6000 (c501): (German, Japan Cobas 6000 series of Roche) fully automated clinical chemistry analyzer [33]. Trained and experienced medical laboratory technologists performed the tests.

Two levels of quality control (QC) were performed every 24 hours, once per reagent kit and after each calibration, to evaluate the instrument’s and reagent’s functionality. Additionally, the results of QC were assessed using the Levey–Jennings chart (Wesgard rules). The calibration method has been standardized against the WHO International Standard NIBSC code: 03/178, 1st International Standard (IS) NIBSC (National Institute for Biological Standards and Control) “Reagent for Ferritin (human liver)” 80/602, and Reference preparation of the IRMM (Institute for Reference Materials and Measurements) BCR470/CRM470 (RPPHS-Reference Preparation for Proteins in Human Serum) for serum ferritin and serum hsCRP, respectively. Calibration was performed as per the standard operating procedures (SOPs). In addition, well-trained medical technologists measured hemoglobin concentration from capillary blood using a portable HemoCue Hb 301®, the gold standard for fieldwork [33].

### Data quality assurance

Two training days were given for data collectors, laboratory professionals, and supervisors before the pre-test. The questionnaire pre-test was conducted on 10% of the sampled pregnant women in a district that was not included in the main study; appropriate adjustments were made based on the results. Supervisors closely managed data collection, checking the data daily before entry. The investigators administered all data collection activities. In addition, laboratory analysis quality assurance at the National Reference Laboratory at EPHI was monitored. The EPHI laboratory is accredited by the Ethiopian National Accreditation Office (ENAO), conducting tests following ISO 15189:2012, Quality and Competence Medical Laboratory Requirements (accreditation no. M 0025). Well-trained and experienced laboratory professionals strictly followed standard operating procedures for all parameters.

### Data processing and analysis

Data were double entered using Epi-data 3.1. Data were cleaned, coded, checked for missing and outliers, and analyzed using Stata 14 (College Station, Texas 77,845 USA). The outcome variable (iron status) was dichotomized as ID (coded as 1) and normal (coded as 0). Log-binomial regression and linear regression analyses were fitted to identify predictors of ID. Next, the binary analysis variables with a *p* < 0.25 were entered into the adjusted log-binomial models. Results were presented using the crude relative risk (CRR) and adjusted relative risk (aRR). The goodness-of-fit was assessed using the Pearson chi-square and deviance tests, with the statistical significance level at alpha = 5%. The explanatory variables were examined for multi-collinearity before taking them into the multivariable model using a correlation matrix for the regression coefficients, the standard errors, and the variance inflation factor value. Hemoglobin values were adjusted for altitude per the Center for Disease Prevention and Control (CDC) recommendation [33].

Since infection can lead to an elevation in ferritin levels, the higher ferritin cut-off point (SF < 15 μg/L), recommended by the WHO for developing countries, was used to define ID and compensate for the effect of infections. Iron deficiency, moderate iron depletion, and iron sufficiency were defined as SF less than 15 μg/L, 15–30 μg/L, and > 30 μg/L [33]. Serum CRP levels greater than 5 mg/L were considered high CRP [33]. Adjustments were made by raising the ferritin cut-off value for individuals with an infection or inflammation to define deficiency to less than 70 μg/l. Anemia was described as a hemoglobin level of < 11.0 g/dl during the first or third trimester or < 10.5 g/dl during the second trimester [33].

As the detailed description has been given elsewhere in a previous paper [32], the wealth index was employed to estimate the economic level of families. The wealth dispersion was generated by applying the principal component analysis (PCA). The index was calculated based on the ownership of latrines, agricultural land and size, selected household assets, livestock quantities, and source of drinking water, a total of 41 household variables. The previous paper [26] described nutritional knowledge and attitudes toward consumption of an iron-rich diet using the Likert scale applying the PCA; the factor scores were totaled and classified into tertiles. Women’s autonomy was evaluated using seven validated questions adopted from the Ethiopian Demographic Health Survey [34]. For each question, the response was coded as “one” when the decision was made by the woman alone or jointly with her husband, or “zero” otherwise.

### Ethical considerations

This study was conducted in agreement with the Declaration of Helsinki-Ethical principle for medical research involving human subjects [35]. The proposal was approved by the Institutional Health Research Ethics Review Committee (IHRERC) of the College of Health and Medical Sciences, Haramaya University (ref No: IHRERC/266/2020). Written informed consent was obtained from all participants and legally authorized representatives “of minors below 16 years of age and illiterates,” and confidentiality was maintained by excluding all personal identifiers.

## Results

### Socio-demographic conditions of the participant

**Fig. 1:**
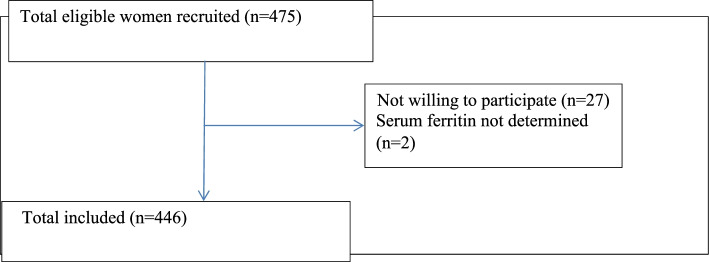
Flowchart of the study

Out of 475 eligible pregnant women, the study included 446, yielding a 93.89% response rate (Fig. 1). Twenty-seven women declined because they were unwilling to provide a venous blood sample, while SF was not analyzed for two women. The mean age of the women was 24.78 (+ 5.20), ranging from 16 to 36. The majority of the respondents could not read or write (73.77%), were housewives (96.64%), farmers (93%), and had a family size of 1–5 (76.46%). Only 19.73% were in the wealthiest quintiles (Table 1).

**Table 1:** Socio-demographic conditions of the participants in Haramaya district, eastern Ethiopia, 2021 (*n* = 446)

Variable	Frequency(n)	Percentage (%)
Age (years)		
< 18	21	5.61
18–35	398	89.24
> 35	23	5.16
Mean (± SD)	24.78 (5.20)	
Educational level of the woman		
Can’t read or write	329	73.77
Read or write	26	5.83
Formal education	91	20.40
Educational Level of husband		
Can’t read or write	257	57.62
Read or write	61	13.68
Grade 1–8	102	22.87
Grade 9 and above	26	5.83
Occupation of the woman		
Housewives	431	96.64
Merchants	15	3.36
Occupation of husband		
Farmers	418	93.72
Daily labors	28	6.28
Family size		
1–5	341	76.46
> 5	105	23.54
Wealth Index (Quintile)		
Poorest	90	20.18
Poor	90	20.18
Middle	89	19.73
Rich	89	19.73
Richest	89	19.73
Gestational trimester during pregnancy		
First trimester	19	4.26
Second trimester	296	66.37
Third trimester	131	29.37
Iron and folic acid supplementation		
No	303	67.94
Yes	143	32.06

### Prenatal iron status

**Fig. 2:**
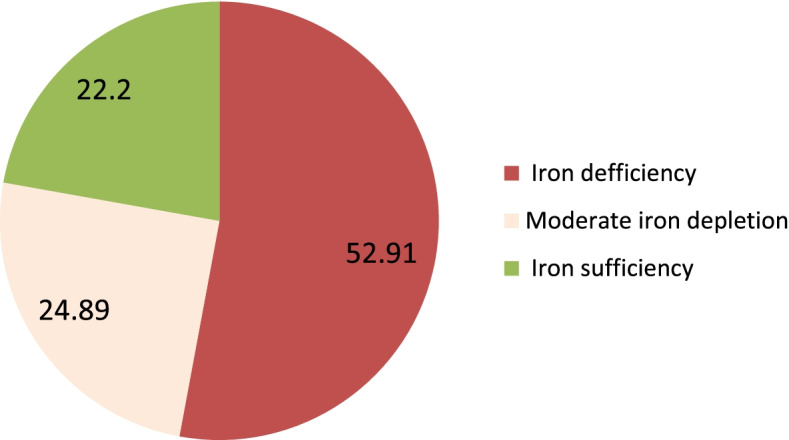
Percentages of iron status of pregnant women in Haramaya District, eastern Ethiopia, 2021

A total of 236 (52.91%) women had ID. Compared to pregnant women with normal iron concentration, women with ID were less likely to have dietary diversity (23.3% vs 63.33%; *p* = 0.03), eat animal source foods (20.76% vs 29.52%; *p* = 0.037) and eat greater than four meals per day (20.76% vs 32.38%; *p* = 0.037). However, compared to pregnant women with normal iron concentrations, those who were iron deficient were more likely to be anemic (52.97% vs 38.10%; *p* = 0.002). Of the 446 women with detectable CRP, 22.87% (95% CI: 19.05–27.05) had levels greater than 5 mg/l, indicating inflammation (Table 2). The median ferritin concentration was 21.08 μg/l (IQR 16.10–23.63) ranging from 1.77 to 134.1 μg/l. As such, 52.91% (95% CI: 48.16–57.63) of the respondents ID. Overall, 24.89 and 22.2% had moderate iron depletion and iron sufficiency, respectively. The mean hemoglobin concentration was 11.25 g/dl (+ 0.05) (95% CI: 11.14–11.35 g/dl), ranging from 7.4 to 151. Thus, the prevalence of anemia and IDA was 45.96% (95% CI: 41.32–50.71) and 28.03% (95% CI: 21.27–32.44), respectively (Fig. 2).

**Table 2:** Serum ferritin concentrations among pregnant women in Haramaya District, eastern Ethiopia, 2021(*n* = 446)

Variables	Total (*n* = 446)	Iron deficiency (*n* = 236; 52.91%)	Normal (*n* = 210; 47.09%)	*P*^a^
Age, Mean (± SD)	24.78 (5.20)			0.160
< 18	5.61	125.0	6.19	
18–35	89.24	91.53	86.67	
> 35	5.16	3.39	7.14	
Educational level of the women				0.351
Can’t read or write	73.77	76.27	70.95	
Read or write	5.83	5.93	5.71	
Formal education	20.40	17.80	23.33	
Parity				0.538
0	22.87	20.76	25.24	
1–4	65.70	67.80	63.33	
≥ 5	11.43	11.44	11.43	
Gestational age (trimester)				0.690
First	4.26	4.76	3.81	
Second	66.37	64.29	68.22	
Third	29.37	30.95	27.97	
Dietary diversity score (DDS)				0.003*
Low	70.40	76.69	36.67	
High	29.60	23.3	63.33	
Consumption of animal source foods (ASFs)				0.037*
Low	75.11	79.24	70.48	
High	24.89	20.76	29.52	
Meal frequency				0.007*
< 4	73.77	79.24	67.62	
≥ 4	26.23	20.76	32.38	
Anemia				0.002*
No	54.04	47.03	61.90	
Yes	45.96	52.97	38.10	
Iron deficiency anemia (IDA)				< 0.001**
No	71.97	47.03	100	
Yes	28.03	125	0	
C-Reactive Protein (CRP)				0.024*
CRP < 5 mg/l	77.13	72.88	72.88	
CRP ≥ 5 mg/l	22.87	27.12	81.90	

** Statistically significant at *p*- value < 0.001; * statistically significant at *p*-value < 0.05

### Factors associated with iron deficiency

In the crude analysis, history of anemia, unprotected source of drinking water, antenatal care, maternal nutritional status, marital status, educational level of the woman and women’s decision making, skipping meals, and dietary diversity were found to be associated with ID at a *p*-value < 0.25. In the adjusted analysis, however, only dietary diversity score, skipping meals and ANC follow-up remained statistically significant. The risk of ID was more likely among women with low dietary diversity (aRR = 1.36; 95% CI = 1.07–1.72) and who experienced skipping meals (aRR = 1.29; 95% CI = 1.05–1.57) whereas it was significantly lower among women who had antenatal care follow-up (aRR = 0.73 (95% CI = 0.61–0.88) compared with their counterparts, Table 3.

**Table 3:** Factors associated with Iron deficiency among pregnant women in Haramaya district, eastern Ethiopia, 2021

Variables	Iron deficiency		CRR (95% CI)	aRR(95% CI)	*p*-value
	Yes (*n* = 236)	No(*n* = 210)			
Antenatal care					< 0.001**
No	105 (55.51)	59 (71.90)	1	1	
Yes	131 (44.49)	151 (28.10)	0.73 (0.61, 0.86)	0.73 (0.61, 0.88)	
Dietary diversity score					0.011*
High	55 (23.31)	77 (36.67)	1	1	
Low	181 (76.69)	133 (63.33)	1.38 (1.11 1.73)	1.36 (1.07, 1.72)	
History of anemia					0.933
No	67 (28.39)	76 (36.19)	1	1	
Yes	169 (71.61)	134 (63.81)	1.19 (0.97, 1.46)	0.99 (0.79, 1.23)	
Maternal nutritional status					0.097
Normal	114 (48.31)	118 (56.19)	1	1	
Undernutrition^b^	122 (51.69)	92 (43.81)	1.16 (0.97, 1.38)	1.16 (0.97, 1.38)	
Skipping meals					0.014*
No	72 (30.51)	88 (41.90)	1	1	
Yes	164 (69.49)	122 (58.10)	1.27 (1.05, 1.55)	1.29(1.05, 1.57)	
Source of drinking water					0.514
Protected	95 (40.25)	103 (49.05)	1	1	
Unprotected	141 (59.75)	107 (50.95)	1.18 (0.99, 1.42)	0.93 (0.76, 1.15)	
Women decision making					0.192
No	226 (95.76)	189 (90.00)	1	1	
Yes	10 (4.24)	21 (10.00)	0.59(0.35, 0.99)	0.71 (0.42, 1.19)	
Marital status					0.503
Married	229 (97.03)	198(94.29)	1	1	
Others	7 (2.97)	12 (5.71)	0.69 (0.38, 1.25)	0.82 (0.45, 1.48)	
Educational level of the woman					0.513
No formal education	192 (82.20)	161 (76.67)	1	1	
Literate	42 (17.80)	49 (23.33)	0.84 (0.66, 1.078)	0.93 (0.74, 1.17)	

*CRR* Crude Risk Ratio, *aRR* adjusted Risk Ratio

** Statistically significant at *p*-value < 0.001; *statistically significant at *p*-value < 0.05

Undernutrition^b^ is defined as pregnant women having MUA < 23 cm

### The concentration of serum ferritin

In the multivariable analysis, we observed that prenatal SF levels among those with low diversified diets was significantly lower (by 0.17 μg/l) than those who had a high diversified diet. Additionally, women who received antenatal care had 0.21 μg/l greater SF concentration compared to their counterparts (Table 4).

**Table 4:** Predictors of ID among pregnant women in Haramaya District, eastern Ethiopia, 2021 (*n* = 446)

Variables	Unstandardized Coefficients	Standardized Coefficients	t	*p*-value
Constant	–	1.677427	2.83	0.005
Mid-upper arm circumference (MUAC) (0= > 23,1 = < 23)	.0319544	.0209064	1.01	0.314
Dietary diversity score (DDS)(0 = Low,1 = High)	−.2263246	.1715377	−1.99	0.047
Marital status (0 = others,1 = married)	.2879749	.2236628	1.20	0.233
Antenatal care follow up (0 = No,1 = Yes)	.2564969	.0758	2.56	0.011
Women decision making (0 = No,1 = Yes)	.2359853	.1476043	0.98	0.327
Educational status of pregnant women (0 = literate,1 = Illiterate)	.2133248	.1152344	1.17	0.242
Drinking water (0 = unprotected,1 = Protected)	−.1533354	0156889	−0.19	0.849

*MUAC* Mid-upper arm circumference, *DDS* Dietary diversity score, *ANC* Antenatal care, *PW* Pregnant women

## Discussion

In this study, we reported iron status and deficiency among pregnant women in Haramaya District, eastern Ethiopia using serum ferritin concentration. We found that half (52.91% (95%CI: 48–57) of the pregnant women were ID, most frequently in the second trimester, even with the universal provision of iron and folic acid supplementation to all antenatal women in Ethiopia. Moreover, the risks of iron deficiency were higher among women with low dietary diversity and significantly lower among women who had antenatal care follow-up in Haramaya District. To the best of our knowledge, this is the first study to assess the burden of IDA in Ethiopia using SF concentration in a predominantly rural setting. Additionally, since community-based selection was applied in comparison to women presenting to facilities, the use of SF will increase the utility of our findings.

Our findings are higher than previous studies reported in Singapore [5], Ghana [36–38], Thailand [39], and Australia [40]. However, it is consistent with the findings from Palestine [41]. Nerveless it is lower than a study in Ethiopia [25]. The differences in dietary practice, sociocultural variations, and iron absorption may contribute to the higher proportion of iron deficiency. We found nearly half of the women developed anemia during pregnancy, which was comparatively lower than study conducted in Ghana [42] but higher than those reported in Ethiopia [25, 43, 44].

Accumulating evidence indicates that the proportion of anemia due to ID differs by population group, geographical setting, infectious disease burden, and the prevalence of other anemia causes [45]. Our study revealed about 61% of anemia in the pregnant population is due to iron deficiency. This is higher than the study carried out in Gonder, northern Ethiopia [46], which documented that more than half (51.87%) were IDA from anemic pregnant women. However, our result is lower than the findings of a study conducted in Singapore [47] which reported that ID causes 81.3% of anemia. The inconsistency could be due to differences in the study design (facility-based was used in Gonder), study setting, and sociocultural variations. It is likely that most anemic women in our study already had iron deficiency anemia during pregnancy. Since anemia is a late manifestation of iron deficiency, our findings are not surprising.

As expected, we observed women with low DDS were more likely to be iron deficient; this agreed with reports from Thailand [39], India [48–50], and Ghana [37]. This could be due to inappropriate dietary practice or iron absorption ability. We also found that ANC visits reduced the risk for ID in pregnancy, which agreed with a study in Thailand [39] and Ghana [51]. In our research, skipping meals during pregnancy was statistically significant with ID. A similar association was reported in Côte d’Ivoire [52]. Iron deficiency is the most common nutritional anemia that occurs due to poor eating habits. Therefore, the deficiency of essential nutrients in the body may cause ID [53].

The strength of our study includes following strict aseptic techniques during blood drawing, transportation, and processing of blood samples. In addition, we used SF and CPR, which has a high sensitivity for measuring iron status, to avoid underestimation. However, some limitations should also be considered when interpreting our findings. The cross-sectional nature of the data limits causal inference between ID and its correlates.

## Conclusion

We found that half of the pregnant women in our study area had ID. Additionally, a quarter of them were prone to IDA. The main risk factors for prenatal ID were no antenatal care, a low diversified diet, and skipping meals. We believe in improving iron levels of pregnant women, a comprehensive strategic approach is needed. The first step is improving animal source foods intake and diversification of diets. Next, strengthening nutritional counseling and services, including providing supplements during prenatal care, is essential. Increasing the knowledge of a balanced diet, the benefits of nutritious foods, especially iron-rich foods, and the importance of a healthy lifestyle may contribute to preventing ID. Lastly, focused attention on encouraging ANC attendance and better compliance to ANC interventions by pregnant women is strongly recommended. Health professionals and care providers should provide complete information and advice about appropriate ANC care for every pregnant woman. Additionally, a prospective study comparing maternal and perinatal outcomes among these spectra—iron depletion, ID, and IDA—is essential for understanding the importance of each condition on maternal and perinatal mortality and morbidity.

## Data Availability

All data are available within the manuscript. Additional data can be obtained from the corresponding author on a reasonable request.

## Abbreviations

ANC: Antenatal care
ASF: Animal source foods
DDS: Dietary diversity score
EDHS: Ethiopian Demographic and Health Survey
IDA: Iron deficiency anemia
HDS-HRC: Haramaya Demographic Surveillance and Health Research Center
PCA: Principal Component Analysis
WHO: World Health Organization
